# The elimination of healthcare user fees for children under five substantially alleviates the burden on household expenses in Burkina Faso

**DOI:** 10.1186/s12913-015-0957-2

**Published:** 2015-08-08

**Authors:** Mahaman Mourtala Abdou Illou, Slim Haddad, Isabelle Agier, Valéry Ridde

**Affiliations:** Cellule d’Analyse et de Prospective en Développement (CAPED), Cabinet du Premier Ministre, République du Niger, 28, Avenue du Mounio, BP 13.568, Niamey, Niger; Département de médecine sociale et préventive, Faculté de Médecine, Université Laval, Québec, Canada; Centre de recherche du CHU de Québec, 1050 chemin de Ste-Foy, Québec, QC G1S-4L8 Canada; University of Montreal Hospital Research Centre (CRCHUM), Saint-Antoine Tower, 850 Saint-Denis St., Montreal, QC H2X 0A9 Canada

## Abstract

**Background:**

Since September 2008, an intervention has made it possible to provide free care to children under five in public health facilities in two districts of Burkina Faso. This study evaluated the intervention’s impact on household expenses incurred for services (consultations and medications) to the children targeted.

**Methods:**

The study is based on a survey of a representative panel of 1,260 households encountered in two waves, one month before and 12 months after the introduction of the intervention. The questions explored the illness episodes of all children under five in the 30 days before each wave. The analysis of health expenses incurred during an illness episode distinguished between total expenses and those incurred in public health facilities (charges for services and medications). Analyses based on multilevel simultaneous equation models were used to estimate the probability of spending and the amount spent, in a context where a large number of observations returned a count of zero.

**Results:**

The burden on household expenses was greatly alleviated under the intervention. Average expenditure dropped from US$11 per episode of care to less than US$2 after the intervention was implemented. The risk of incurring an expense at a public health facility was reduced by two-thirds. The facility users’ savings were primarily related to medication purchases. In rural areas, where barriers to access health services are more acute, both poor and non-poor families benefited from the intervention. The probability of spending on medications dropped dramatically for both the poor and the non-poor under the exemption (−75 % vs.–77 %), and the reduction in expenses for medications generated by the intervention was comparable for both groups in relative values (−86 % vs.–89 %).

**Conclusion:**

User fees abolition at the point of service substantially alleviated the burden on household expenses. The intervention benefited both poor and non-poor families and provided financial protection.

## Background

The international movement toward universal healthcare coverage [1] has recently prompted many African countries to undertake interventions aimed at increasing health services use. These interventions primarily target pregnant women and children under the age of five years, in the context of the Millennium Development Goals [2]. Of the many barriers encountered by healthcare users [3, 4], the financial one has been the primary focus of decision-makers and researchers in recent years [5]. In fact, the great majority of interventions to eliminate the financial barrier have been very effective in achieving the intended objective of increasing the use of healthcare services across the board, and especially among the worst-off [6, 7]. However, in contexts where the implementation of these interventions is often chaotic and where health system governance is not always perfect [8, 9], there is still some risk that the increase in health services use would not be accompanied by financial protection. Yet financial protection is one of the major objectives of health systems [1, 10]. Using the system should not make the poor even poorer [11, 12]. In effect, “*access to services alone, without protection from financial ruin, provides an empty promise”* [10] (p.1932). Moving toward universal health coverage should mean that out-of-pocket (OOP) expenses for health interventions would become “*zero or close to zero”* [10] (p. 1932). The World Bank has just set a new objective: “*By 2030, no one should fall into poverty because of out-of-pocket health care expenses”* [13]. In fact, a recent review showed that interventions aimed at universal health coverage do provide financial protection to populations [14].

Several studies have shown that, despite their low population coverage in African countries, community-based health insurance and mutual health organizations were able to protect their members financially [15, 16]. It is particularly surprising, however, that the substantial wave of user fees abolition interventions in Africa [8, 9] has been accompanied by very little research to evaluate their impacts on the users’ financial protection. There have been several studies on the financial protection of women in childbirth in the context of such interventions in Burkina Faso and Mali [17–20]. We found no study related to the financial protection of households in which children under five benefited from user fees exemptions. The most recent systematic review (Cochrane) on the subject confirmed the lack of evidence in this area [7]. For example, published studies on interventions providing free care to children in Ghana, Mali, Niger, Rwanda, and Sierra Leone did not measure the impacts on the financial protection of households [21–25]. In a rural area of South Africa, one study showed the protective effects of the primary care user fees exemption policy [26]. In Uganda the user fees exemption policy targeted the entire population, not only children. It had no impact on financial protection [27]. Moreover, the rise in health services use and the difficulties involved in implementing the intervention undoubtedly explain why health expenses increased under the exemption [28].

In Burkina Faso, only 50 % of sick children use health facilities [29]. Even though numerous reforms have helped to improve the healthcare system over the past 20 years, the population still faces a major financial barrier [30]. Consequently, in September 2008 an intervention was implemented in two districts (Dori and Sebba) of a Sahel health region where the health needs were greatest. Its aim was to improve access to care in health facilities for the most vulnerable groups in the population: children, pregnant women, and the indigent. Beginning in September 2008, all healthcare services, including medications, consultations, and evacuations to hospitals, were exempted from point-of-service user fees. The intervention was piloted by a German NGO and funded by the European Union. Primary care health facilities providing free care for these beneficiaries were reimbursed by the NGO on a monthly basis. To maintain quality of care, supervision and training were provided by NGO personnel in collaboration with leaders of the district management team, an approach that has been shown to be effective [31]. Various actions were also carried out to make the population aware of the free services being provided, to inform communities, and to strengthen the capacities of health facilities’ management teams. The intervention continued without interruption from the time of its launch. The intervention was entirely integrated into the healthcare system, except for the fact that these services were reimbursed by ECHO (European Community Humanitarian Aid Office). For example, the State remained responsible for drug distribution channels and health personnel in accordance with the usual conditions for healthcare system operation. At the same time, activities were organized to mobilize communities and inform the population. This intervention proved to be very effective and equitable in improving the use of services [32, 33]. The objective of this paper is to present the impacts of the intervention on households’ burden for healthcare costs for children under five using health facilities covered by the intervention.

## Methods

The study is based on a population survey carried out in one of the two intervention districts (Dori district). The population of the district was about 290,000 when the study began in 2009. Children under five represented about 20 % of the population. The district included 17 health and social promotion centres (CSPSs) linked to the regional hospital (CHR) [34]. The survey involved a representative panel of 1,260 households. Households were randomly selected using a two-stage sampling approach adapted from the methodology of the World Health Organization Expanded Programme on Immunization (EPI) Cluster Survey Design [35]. Based on the enumeration areas defined by national census, 48 clusters of households were first randomly selected in the district, with the clusters proportional to the size of the enumeration areas. Then 30 households per cluster were selected based on the adapted EPI sampling scheme.

Two rounds were conducted (2008 & 2009) at the same period of the year to limit seasonal variations in morbidity and in households**’** financial liquidity. The survey included questions on each household’s composition, living conditions, and healthcare practices. All children under five were identified. Before the interviews, consent was obtained from either the mothers or other adults looking after the children in the household. For each child, the surveyors inquired about any occurrences of illness episodes during the preceding 30 days. The characteristics of the illness episode, the use of services, and the costs associated with those services were provided by either the mother or the person best positioned to provide this information. Episodes were then documented: date, type and duration of symptoms, perceived seriousness of the illness, use of healthcare services and particularly visits to public health facilities (health centres or hospitals), and both medical expenses (services and treatments) and non-medical expenses incurred for the care provided. Household socioeconomic status was estimated on the basis of household consumption. The households were divided into income quartiles for each of three brackets representing distance between home and health facility (0–4 km; 5–9 km; 10 km and over).

Before the intervention, the distribution of healthcare expenses already showed a large number of observations with a count of zero, since in many episodes there was no payment made for services or medications (for example, in cases of self-medication). The zero value mass increased in 2009 as a result of the introduction of free services at public health facilities. The literature suggests a variety of methods for taking into account these distinctive distributions [36–38]. We used simultaneous equation modelling [39].

We performed three simultaneous two-equation multilevel models. The first equation modelled the probability of spending. The second was a linear regression on the amounts spent when expenses were incurred during the illness episode: total amount (model 1), medical services (model 2) and medications (model 3). The table in Appendix 1 presents the dependent and independent variables used. Independent variables had to do with the child, the family, the living environment, and the services used during the episode of illness (Appendix 1). Because of multicollinearity, particularly between socioeconomic variables, and for the sake of parsimony, the final models included only those variables significantly associated with the selection variable or the outcome. The models retained were those presenting the best fit as estimated by goodness-of-fit tests for maximum likelihood-based estimates (Loglikelihood, AIC & BIC criteria). The variables included in the final models were the year of observation, the symptoms, the child’s age, the household’s income, rural or urban residence, and the type of services used during the episode. Two interaction terms were added between the year and the use of a public health facility, and between the year and household poverty level. All analyses were performed using MLWin software [40], which supported the development of multilevel simultaneous equation models that took into account the hierarchical structure of the data (episodes observed among children in a household panel in 2008 and 2009). Stata 11 software was also used to perform statistical significance testing and difference in differences analysis.

The research was approved by the research ethics committees of the Ministry of Health in Burkina Faso and the University of Montreal Hospital Research Centre in Canada.

## Results and discussion

Table 1 presents the main characteristics of the panel and of the illness episodes of the children. There were 1,251 households surveyed in 2008; attrition was minimal (1,191 households in 2009) and no significant difference was observed in the characteristics of the respondent households between 2008 and 2009. This being a Sahelian region, the vast majority of the households lived in rural settings. The typical household was headed by a man and had, on average, two children under five; the mother’s level of education was low. At baseline, one household in five reported that a child had been ill in the preceding 30 days; that proportion was slightly lower in 2009, but the difference was not significant. The number of sick children naturally exceeds the number of households reporting a sick child. The characteristics of the sick children in both years were comparable. Table 1 also shows there was a substantial and significant increase in the use of health facilities and hospitals in the post-intervention period. After the intervention was introduced, more than one episode in two resulted in a visit to a public health facility, whereas before the intervention, that ratio was just over one in three. A detailed analysis of the intervention’s effects on the use of services is beyond the scope of this paper and has been presented in another article [32].

**Table 1:** Household characteristics and description of the sample

		Year		Difference
		Pre-intervention (2008)	Post-intervention (2009)	2009–2008^d^
1. Households surveyed (n=)		1251	1191	
	Households surveyed in 2008 *and* revisited in 2009	1182 (94 %)		
	Located in a rural area	1091 (87 %)	1039 (87 %)	0
	Woman-headed household	37 (3 %)	34 (3 %)	0
	Mother never went to school	782 (63 %)	714 (60 %)	−3
	Household size			
	*Mean (Standard Deviation)*	7.7 (4.1)	7.9 (4.4)	0.2
	*Median (IQR)*	7 (4)	7 (5)	0
	Children under five per household			
	*Mean (Standard Deviation)*	2.2 (1.1)	2.8 (1.1)	−0.02
	*Median (IQR)*	2 (2)	2 (2)	0
2. With least one episode of illness for a child^a^ (n=)		281	198	
	As a proportion of households surveyed	22 %	17 %	−5
	Located in a rural area	260 (93 %)	184 (93 %)	0
	Poor	122 (43 %)	93 (47 %)	−4
3. Sick children (n=)		321	220	
	Girls	153 (48 %)	102 (46 %)	−2
	Living in a rural area	299 (93 %)	204 (92 %)	−1
	From a poor household	182 (56 %)	117 (53 %)	−3
	Whose mother never went to school	297 (92 %)	204 (92 %)	0
4. Episodes of illness (n=)		322	221	
	Including episodes considered serious by the respondent	96 (30 %)	58 (26 %)	−4
	Primary symptoms			
	*Respiratory*	25 (8 %)	26 (12 %)	4
	*Digestive*	178 (55 %)	118 (53 %)	−2
	*Fever*	94 (29 %)	66 (30 %)	1
5. Episodes of illness resulting in an expense (n=)		163	68	
	As a % of illness episodes reported	51 %	31 %	−20**
	From rural households	92 %	84 %	−8*
	Total spending per episode (when cost >0) in US$^b^			
	*Average cost and (Standard Deviation)*	8.87 (9.77)	4.41 (5.65)	−4.46***
	*Median cost (IQR)*	5.92 (9.35)	2.17 (3.32)	−3.75***
6. A public health facility was used during the episode^c^		121	124	
	As a % of illness episodes reported	38 %	56 %	18**

^a^Recall period = 30 days

^**b**^Average rate of exchange 2008–2009: US$1 = 460 F CFA. Source: http://www.bankofcanada.ca/rates/exchange/10-year-converter/?__utma=1.1843689861.1395416059.1395416059.1395416059.1&__utmb=1.1.10.1395416059&__utmc=1&__utmx=-&__utmz=1.1395416059.1.1.utmcsr=(direct)|utmccn=(direct)|utmcmd=(none)&__utmv=-&__utmk=130907791

^c^Health centre or hospital. Use of services not exclusive: one household may have used health services more than once for the same illness episode

^d^Differences expressed as percentage points, except for costs, where the difference expresses the nominal difference

* P <0.05; ** P <0.01; *** P <0.001

**Fig. 1:**
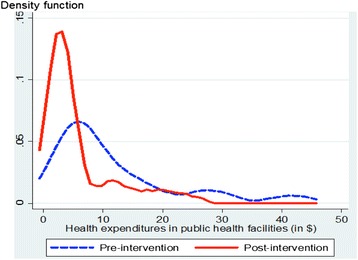
Distribution of expenses incurred by users of public health facilities before and after implementation of the intervention. Sample of episodes including an expense incurred at a health centre

In 2009, families incurred expenses only in one-third of cases (35 %), whereas before the intervention almost all episodes of care in a public health facility resulted in an expense (94 %) (Table 2). In the majority of cases, the charges were for medications or consumables provided to the child. Fewer than one episode in six incurred an expense for care and services provided by health personnel. With the intervention, the median expense in a public health facility was reduced by more than half for families who paid for their children’s care. Figure 1 shows the distribution of expenses incurred during an episode of care for services received at a health facility, before and after the intervention’s implementation. Comparison of the density functions shows a shift toward the origin and a narrowing of the curves during the intervention period (Table 2). Therefore, during the intervention period, expenses were incurred less often for episodes of illness, and when there were expenses, they were substantially lower.

**Table 2:** Healthcare expenses incurred by users of public health facilities before and after implementation of the intervention^a^

	Pre-intervention (2008)	Post-intervention (2009)	Savings 2009–2008
1. All episodes in a public health facility	121	124	
Resulting in an expense	114 (94 %)	43 (35 %)	(− 63 %)
Average amount spent (SD)	11.6 (10.4)	5.7 (6.0)	5.9***
Median amount spent (IQR)	8.0 (10.2)	3.5 (4.6)	4.6***
2. Payment for consultation	56	9	
As a % of episodes	46 %	7 %	(−85 %)
Average amount spent (SD)	2.4 (3.4)	3.0 (3.1)	-
Median amount spent (IQR)	1.1 (1.7)	2.6 (2.7)	-
3. Payment for medication	99	15	
As a % of episodes	82 %	12 %	(−85 %)
Average amount spent (SD)	7.5 (7.1)	5.2 (5.9)	-
Median amount spent (IQR)	4.8 (5.9)	3.3 (2.3)	-
4. Payment for hospitalization	11	4	
As a % of episodes	9 %	3 %	(−67 %)
Average amount spent (SD)	6.5 (5.8)	1.9 (0.2)	4.6*
Median amount spent (IQR)	4.8 (9.8)	1.9 (0.3)	-
5. Payment for other services	14	8	
As a % of episodes	12 %	6 %	(−50 %)
Average amount spent (SD)	2.3 (3.2)	10.2 (19.9)	-
Median amount spent (IQR)	1.5 (1.1)	3.3 (7.2)	-

^a^Sample: Illness episodes resulting in a visit to a public health facility. Average rate of exchange 2008–2009: US$1 = 460 F CFA

* P <0.05 ; ** P <0.01 ; *** P < 0.001

After adjusting for confounding variables, the models showed that the risk of incurring an expense in a health facility was reduced by 60 % (Table 3). The savings realized by users of public health facilities were substantial as the adjusted average cost of an illness episode dropped from US$11 per episode to less than US$2 during the intervention period, an average savings per episode of illness of nearly US$9. As expected, the adjusted average cost of an illness episode that did not involve the use of a public health facility remained unchanged. This cost is relatively low (less than US$0.30), and is essentially incurred when medications are purchased in markets or stores. On the other hand, the risk was unchanged when the child was not brought to a health centre or hospital.

**Table 3:** Effects of the intervention on total health expenditures during an episode of illness for users and non-users of a public health facility^a^

		Episode of illness			
		With use of a public health facility		Without use of a public health facility	
Probability of incurring an expense during the episode^b^		Estimate	CI	Estimate	CI
	Pre-intervention propensity (P1)	0.92	[0.86; 0.96]	0.27	[0.22; 0.34]
	Post-intervention propensity (P2)	0.37	[0.28; 0.45]	0.29	[0.21; 0.37]
	P_2_ – P_1_	−0.55	[−0.65; −0.45]	0.02	[−0.09; 0.13]
	P_2_ / P_1_ – 1	−60 %		7 %	
Total spending (in US$) in cases of expenses^c^		Estimate	CI	Estimate	CI
	Total cost pre-intervention (C1)	11.01	[10.00; 12.03]	0.65	[−0.15; 1.41]
	Total cost post-intervention (C2)	1.92	[0.82; 2.97]	0.55	[−0.64; 1.74]
	C2 – C1: absolute savings	−9.09	[−10.58; −7.60]	−0.10	[−1.31; 1.11]
	C2 / C1 – 1: relative savings	−83 %		−15 %	

^a^Values predicted by simultaneous equation modelling. The sample consists of all illness episodes

^b^First equation (probit): Probability of incurring an expense. Adjustment variables: year, use of health facility, use*year interaction, location of residence, household income, age of child

^c^Second equation (linear regression): Amount spent. Adjustment variables: year, use of health facility, use*year interaction, location of residence, household income, age, primary symptom, use of traditional practitioner, use of other providers

The intervention’s impact on expenses incurred by the poor and the non-poor for visits to health facilities is presented in Table 4. Because of the very small number of episodes observed in urban health facilities (10 in 2008 and 12 in 2009), the analysis was conducted only on the subsample of households living in the rural settings. The first model relates to expenses for services, whereas the second model relates to expenses incurred for medications purchased. Columns 1 to 4 refer to the first equation, modeling the propensity to incur an expense when visiting a health facility (probit model). Columns 5 to 8 refer to the second equation, modeling the amount of the expenditure when an expense was incurred.

**Table 4:** Effects of the intervention on expenses incurred by users of public health facilities by household income Sample: Illness episodes resulting in a visit to a public health facility in rural areas

		Eq1: Probability of incurring an expense^b^				Eq2: Amount spent when expenses were incurred (in US$)^c^			
		Pre-intervention (2008) (1)	Post-intervention (2009) (2)	Difference(3)	Difference in differences (4)	Pre-intervention (2008) (5)	Post-intervention (2009) (6)	Difference(7)	Difference in differences (8)
Model 1^a^: Expenses for services									
	Poor	0.41	0.15	−0.26		0.95	0.33	−0.62	
		[0.28; 0.54]	[0.07; 0.25]	[−0.37; −0.15]		[0.43; 1.47]	[−0.16; 0.85]	[−1.35; 0.11]	
	Non-poor	0.60	0.09	−0.51		1.39	0.34	−1.05	
		[0.49; 0.71]	[0.04; 0.17]	[−0.60; −0.42]		[0.97; 1.86]	[−0.10; 0.83]	[−1.69; −0.40]	
	Difference	−0.19	0.06		0.25	−0.44	−0.01		0.43
		[−0.30; −0.08]	[−0.03; 0.15]		[0.17; 0.33]	[−1.12; 0.25]	[−0.70; 0.68]		[−0.55; 1.40]
Model 2^a^: Expenses for medication purchases									
	Poor	0.91	0.16	−0.75		6.48	0.88	−5.60	
		[0.82; 0.97]	[0.07; 0.28]	[−0.83; −0.67]		[4.94; 8.21]	[−0.47; 2.15]	[−7.70; −3.49]	
	Non-poor	0.88	0.11	−0.77		8.12	0.92	−7.21	
		[0.79; 0.95]	[0.05; 0.20]	[−0.84; −0.70]		[6.75; 9.41]	[−0.50; 2.38]	[−9.18; −5.24]	
	Difference	0.03	0.05		0.02	−1.64	−0.04		1.60
		[−0.04; 0.10]	[−0.04; 0.14]		[−0.05; 0.09]	[−3.76; 0.47]	[−1.99; 1.93]		[−1.28; 4.50]

^a^For each model, values predicted by simultaneous equation modelling (^b^ and ^c^)

^b^Predicted values, probit model

^c^Predicted values, linear regression model. Average rate of exchange 2008–2009 : US$1 = 460 F CFA

Adjustment variables in each equation (^b^ and ^c^): year, household income, year*household income, age, location of residence

The results show that, after adjusting for confounding factors, the probability that the poor would have to pay for a consultation was 41 % before the intervention. Once the intervention was in place, that risk was only 15 %, such that the benefit, expressed as difference in risk, is 26 percentage points (Dif. ⊂ [−0.37; −0;15]). The non-poor also benefited significantly from the intervention; the adjusted risks were 60 % and 9 % before and after the intervention, respectively, for a relative gain of 51 percentage points, also significant. The model also suggests that the risk of having to pay for a consultation before the intervention was significantly and markedly greater among the non-poor (60 %) than the poor (40 %), a result that can be explained by the latter’s more limited contributive capacity. After the intervention’s implementation, this risk was low for both groups, and the gap between the poor and non-poor was no longer significant (Dif. ⊂ [−0.03; 0.15]). However, given their initial conditions and their different progressions, the difference in differences between the two groups is significant and more advantageous for the non-poor (DID = 0.25 [0.17; 0.33]).

The situation is different with respect to medications. In the vast majority of cases, a visit to a health facility before the intervention ended in a payment for medications, whether the patient was poor (*P* = 0.91) or not (*P* = 0.88). The intervention’s effect was spectacular and significant for both the poor and the non-poor. Furthermore, the risks were similar for both groups before (D = 0.03) and after (D = 0.05) the intervention, as were their respective gains (DID = 0.02).

Before the intervention, the mean amount spent for a consultation was slightly less than US$1 for children from poor households and around US$1.4 for the non-poor (difference not significant). In both cases, the reduction observed after the intervention was not significant, nor was the difference in differences. In other words, we cannot conclude that the intervention led to a reduction in the cost of consultations for residual households that were not exempted, that is, 15 % of poor households and 9 % of non-poor households.

Medications were by far the most expensive expenditure item; the average spending on them before the intervention was six to seven times greater than the spending on consultations. In fact, the savings realized, both by the poor and the non-poor, had mainly to do with the cost of medications. These expenses were reduced by US$5.6 for the poor and US$7.2 for the non-poor, representing a reduction in spending of 86 % and 89 %, respectively, compared to the pre-intervention period. Both differences are highly significant. The differences in differences suggest the intervention did not benefit one group more than another.

### Study strengths and limitations

To our knowledge, this is the first longitudinal study on this matter conducted in Africa [7]. The approach used made it possible to chart the evolution in health services use in a household panel from shortly before to one year after the intervention’s implementation. Fresh evidence was provided on the impact of the user fees removal, taking into consideration the distinctive distribution of health expenses and the hierarchical structure of the data. Equity analyses using differences in differences made it possible to compare the effects of the intervention across social groups and to verify whether there was any risk that the intervention might benefit only the children of well-off households.

Three limitations should be noted in this study. The first relates to the design. It was not possible to use an experimental design and there was no external group that was sufficiently comparable to the study context which could be paired with the intervention site. The research design is thus potentially vulnerable to historical biases [41] that cannot be definitively ruled out. However, there was very little attrition in the panel, and the risks of history and maturation biases were contained by the short observation window and close monitoring of the presence of any other events that could have tangibly affected service provision or health needs. The use of out-of-pocket expenses rather than “catastrophic” health expenses [12] as an indicator of financial protection could be seen as another limitation. However, we felt that catastrophic expenses would have likely underestimated the economic effects of the intervention, as it is unlikely in this context that households would incur expenses that could negatively affect their well-being for the sake of a child’s health. In addition, in this Sahelian context where consumption is largely based on households’ self-production and family solidarity helps to broaden households’ capacity to pay [42], valid thresholds of catastrophic expense appeared difficult to establish. The third limitation lies in the size of the illness episode sample. While the panel originally included 1,260 households, in the end there were only 281 episodes of illness involving children under five to analyze in the pre-intervention period and 198 in the intervention period. This may have limited our ability to determine the effectiveness of the intervention.

### Effects of the intervention and policy implications

There is now substantial evidence, with regard to both pregnant women and children, that removing point-of-service user fees increases healthcare services use [6, 7, 18, 32]. The present study confirms that eliminating point-of-service user fees for households with sick children appears to be an effective strategy for reducing households’ financial burden.

Access to health services increased for the poor, and the removal of user fees reduced their financial burden substantially and significantly. The average savings for the poor in rural areas were around US$6 for a child’s episode of illness, or the equivalent of 12 days of basic living expenses in a country where 46 % of the population lives below the poverty line [43]. Qualitative studies of this intervention in Burkina Faso have also suggested that making services free has contributed to women’s empowerment by removing the need for them to obtain money from the head of household for healthcare for themselves and their children [44]. The results of our study show that the user fees exemption did not benefit primarily the poor, neither with respect to the risk of having to pay for services provided in health facilities, nor in terms of the amounts incurred for such expenditures. Rather, they suggest that the various strata of the population, whether poor or not, benefited from this intervention—a significant benefit in this poor country. These results reinforce the findings of other studies showing that fears that the less poor will benefit more from public health interventions than will the worst-off are not really well-founded in this context [45].

The government also drafted a national social protection policy in 2012, in which it recommends eliminating user fees for those same target populations as well as for the indigent [46]. Thus, there appears to be a certain political will to support the elimination of user fees and the transition to universal health coverage, even though some key decisions have yet to be taken. While this intervention has been shown to have positive effects, any scaling-up of this type will require strong political will and appropriate means of action, both of which are essential factors for successful health policies [47]. In this event, we believe at least three inter-related challenges should be considered. The first challenge is to maintain the quality of care. A study has shown that the intervention presented here was successful in maintaining the quality of care [31]. However, it will be important to ensure that the support activities and medical supervision continue after the NGO has gone, which falls within the State’s usual responsibilities. The second challenge relates to equity. Poor targeting of the worst-off has always been a shortcoming of health policies in Burkina Faso. Specific measures need to be organized so that they can benefit even further from this type of intervention, notably by receiving help to access means of transportation so they can get to health facilities. The last challenge involves fidelity of implementation. Interventions are often implemented with limited adherence to initial plans [48, 49]. In some cases, persons who were supposed to have been exempted from user fees have reported being charged certain expenses [19]. In our study, one out of six mothers reported having had to pay for services in a public health facility after the exemption was implemented (Table 2). Qualitative studies would help to shed light on this implementation gap. To ensure such studies are properly carried out, it will be important not only to monitor the interventions carefully (monitoring, evaluation, research), but also, and especially, to ensure the necessary financial and material resources are available. Health facilities need to be reimbursed for free services provided, and medical inputs, in particular, must be available, which remains a major challenge for many countries [28, 49].

## Conclusion

This study confirms that point-of-service user fees exemptions substantially alleviate the cost burden for health services users. All the different social groups, including the poor, have benefited greatly from this intervention, which is good news with respect to the movement toward universal health coverage and equity. For greater effectiveness and improved equity, however, it will be important to ensure that free care is actually available to everyone and to supplement the interventions with actions aimed at reducing inequalities in access to care. Beyond the elimination of user fees, for which there is now strong evidence of benefits, governments will also need to adopt more comprehensive policies to achieve universal health coverage [1, 10] and to provide effective and lasting protection against impoverishment related to illness.

## Appendix

**Table 5:** Dependent and independent variables

Variables	Type	Variable specifications
Dependent		
M1: Selection variable		Binomial: 1 if the total amount spent > 0, 0 if not.
M1: Outcome variable		Continuous: Total amount spent during an illness episode in cases of expenses
M2: Selection variable		Binomial: 1 if the total amount spent for medical services > 0, 0 if not.
M2: Outcome variable		Continuous: amount spent for medical services during an episode of illness
M3: Selection variable		Binomial: 1 if the total amount spent for medications > 0, 0 if not.
M3: Outcome variable		Continuous: Amount spent for medication during an episode of illness
Independent		
Year	Intervention	Categorical: 1 if post-intervention period (2009), 0 if pre-intervention (2008)
Setting urban	Contextual	Categorical: 1 if household living in urban area, 0 if not.
Poor (1st quartile)	Family characteristic	Categorical: 1 if poor household, 0 if not.
Age of the child	Child characteristic	Categorical: 1 if the age of the child is between 1 and 5 years, 0 if under one year
Illness: main symptoms	Child characteristic	Categorical: 1 if the main symptoms were digestive; 2 if respiratory; 3 if fever; 4 if other symptoms.
Use of a health facility	Utilisation-child	Categorical: 1 if public health facilities are used for healthcare demand during the illness episode, 0 if not.
Use of a traditional healer	Utilisation-child	Categorical: 1 if healthcare provided by traditional practitioner, 0 if not
Other health seeking behaviour	Utilisation-child	Categorical: 1 if healthcare provided by others healthcare providers, 0 if not
Interaction year*use of a health facility	Intervention*context	Categorical
Interaction year*poverty level	Intervention*family ch.	Categorical
Other variables*		
Sex of the child	Child Characteristic	
Mother never went to school	Family characteristic	
Household size	Family characteristic	
Woman-headed household	Family characteristic	
Distance to the closest facility	Contextual	

*variables initially considered for modelling but not retained in the end because they were not significantly associated with the selection variable nor the cost variable
